# Supplementary material for: Seizure protein 6 controls glycosylation and trafficking of kainate receptor subunits GluK2 and GluK3
Source: EMBO J. 2020 Jun 22;39(15):e103457. doi: 10.15252/embj.2019103457 (PMC7396870; doi:10.15252/embj.2019103457)
Supplement: Supplementary file 1 — Appendix [file EMBJ-39-e103457-s001.pdf]

Appendix

Table of Contents:

Appendix Supplementary Methods

Appendix Figure Legends

Appendix Figure S1: Multiscatter plot of log2 transformed protein LFQ intensities.

Appendix Figure S2: No changes in the total glycome of SEZ6KO neurons were detected.

Appendix Figure S3: SEZ6FL rescues surface levels of GluK2/3 in SEZ6KO neurons.

Appendix Supplementary Methods

Glycome analysis

8M guanidine hydrochloride (GuHCl), 1-hydroxybenzotriazole hydrate (HOBt), 50% sodium hydroxide, super DHB matrix (2-hydroxy-5-methoxy-benzoic acid and 2,5-dihydroxybenzoic acid, 1:9), trifluoroacetic acid (TFA), 28-30% ammonium hydroxide solution and lyophilized recombinant PNGase F from Flavobacterium meningosepticum were obtained from Sigma-Aldrich (St. Louis, MO). HPLC SupraGradient acetonitrile (ACN) was obtained from Biosolve (Valkenswaard, The Netherlands). Dithiothreitol (DTT), ethanol and sodium bicarbonate ($NaHCO_3$) were from Merck (Darmstadt, Germany) and 1-ethyl-3-(3-dimethylaminopropyl) carbodiimide (EDC) from Fluorochem (Hadfield, UK). The peptide calibration standard was purchased from Bruker Daltonics (Bremen, Germany). MultiScreen® HTS 96 multiwell plates (pore size 0.45 µm) with high protein-binding membrane (hydrophobic Immobilon-P PVDF membrane) were purchased from Millipore (Amsterdam, The Netherlands), conical 96-well Nunc plates from Thermo Scientific (Roskilde, Denmark). All buffers were prepared using ultra-pure deionized water (MQ) was generated by the Purelab Ultra, maintained at 18.2 MΩ (Veolia Water Technologies Netherlands B.V., Ede, The Netherlands).

Preparation of released N-glycans from cell pellet

N-glycans were released from twelve biological replicates per condition (0.5 × 10 E6 cells each/25 µl) using a 96-well plate PVDF-membrane based N-glycan release protocol as described earlier(Holst et al., 2016). Briefly, cell pellets were suspended in MQ and sonicated for 30 min. As controls, Visucon pooled human plasma as well as water blanks were used. Denaturation buffer (5.8 m GuHCl and 5 mM DTT) and 25 µl dissolved cell pellet were onto a preconditioned HTS 96-well plates with hydrophobic Immobilon-P PVDF membrane and incubated for 30 min at 60°C. The wells were washed twice with 200 µl mQ with 5 min incubation steps on a horizontal shaker prior to centrifugation and once with 200 µl 100 mM $NaHCO_3$ (1 min, 500 × g). For N-glycan release, 15 µl 100 mm $NaHCO_3$ and 1 mU PNGase F were added per well. After 20 min incubation an additional 15 µl buffer was added. Plates were placed into the incubation device and incubated for overnight at 37 °C. Glycans were recovered into 96-well collection plates by centrifugation (2 min, 1000 × g); eventual residual solution was collected from the membrane and wash 3 times with 40 µl water. Samples were dried for 2 at 45°C in a vacuum centrifuge and finally dissolved in 25 µl water.

MALDI-TOF (/TOF)-MS(/MS) analysis of released glycans

Prior to MALDI-TOF-MS analysis, sialic acids were stabilized in a linkage-specific way by ethyl esterification and amidation (Reiding et al., 2014), purified by cotton-HILIC-SPE, and MALDI-TOF-MS analysis was performed on an UltrafleXtreme (Bruker Daltonics) operated under flexControl 3.3 (Build 108; Bruker Daltonics). Ten microliters of the released glycans were added to 50 µL derivatization reagent (250 mM 1-ethyl-3-(3-(dimethylamino)propyl)carbodiimide and 250 mM 1-hydroxybenzotriazole in ethanol) and incubated for 30 min at 37°C at which 10 µL 28-30% ammonium hydroxide is added for amidation. Sixty microliters of ACN were added and derivatized glycans were enriched by cotton hydrophilic-interaction liquid chromatography (HILIC)−solid-phase extraction (SPE) as described before and eluted in 10 µL water (Selman et al., 2011). Five µL of the enriched ethyl-esterified glycans was spotted on a MALDI target (MTP AnchorChip 800/384 TF; Bruker Daltonics) together with 1 µL 5 mg/mL super-DHB in 50% ACN and 1 mM NaOH. The spots were dried by air at room temperature. For each spot, a mass spectrum was recorded from m/z 1 000 to 5 000, combining 20 000 shots in a random walk patter at 5 000 Hz and 100 shots per raster spot. Prior to the analysis of the samples, the instrument was calibrated using peptide calibration standard (Bruker Daltonics).

Data processing for glycome analysis

For automated relative quantification of the released glycans analyzed by MALDI-TOF-MS, using MassyTools (version 0.1.8.1.) (Jansen et al., 2015), the MALDI-TOF-MS files were converted to text files. Spectra were internally calibrated using glycan peaks of known composition signals with a S/N above nine, covering the m/z range of the glycans. Integration was performed on targeted peaks for visually determined list of glycans, including at least 95% of the theoretical isotopic pattern. Several quality parameters assess the actual presence of a glycan based on the mass accuracy (between -20 and 20 ppm), the deviation from the theoretical isotopic pattern (below 25%) and the S/N (above nine) of an integrated signal. Analytes were included for all samples when present in at least two-thirds of one of the biological replicates. Glycan composition (n = 54) signals were normalized to the total signal intensity.

Appendix Figure Legends

Appendix Figure S1: Multiscatter plot of log2 transformed protein LFQ intensities.

The log2 transformed protein LFQ intensities of all analyzed samples are plotted against each other. Each dot in the scatter plots represents one protein. The Pearson correlation coefficients of each plot are indicated on the left. Scatter plots of samples belonging to the same SUSPECS experiment and related Pearson correlation coefficients are labeled in red (Exp1-Exp3 are independent biological replicates). Samples of the same experiment show a highly reproducible quantification with $R > 0.97$, whereas samples of different experiments correlate with each other with $R > 0.94$.

Appendix Figure S2: No changes in the total glycome of SEZ6KO neurons were detected.

A general N-glycan analysis was performed using lysates of WT and SEZ6KO neurons. N-glycans were released from the protein extracts (Holst et al., 2016) and subject to MALDI-TOF mass spectrometric analysis. N-acetylneuraminic acids (NeuAc) were subjected to linkage-specific derivatization, allowing stabilization of the sialic acid residues and mass spectrometric distinction of sialic acid linkages on the basis of mass shifts induced by ethyl esterification (α2,6-linkage) and lactonization with sequential amidation (α2,3-linkage). 54 glycan structures were identified and quantified, and none of them showed differences between WT and SEZ6KO neurons (plot shows mean ± S.E.M., 11 WT replicates and 12 SEZ6KO replicates from 2 independent biological experiments were used).

Appendix Figure S3: SEZ6FL rescues surface levels of GluK2/3 in SEZ6KO neurons.

Western blot analysis of primary SEZ6KO neurons transduced with either a GFP control or SEZ6FL lentivirus. Surface proteins were biotinylated, enriched with streptavidin beads and blotted for Gluk2/3. Total Gluk2/3 and calnexin were measured in the input. Both total and surface GluK2/3 were normalized for calnexin values (plot shows mean ± S.E.M., n=4, Mann-Whitney *p-value=0.0286).

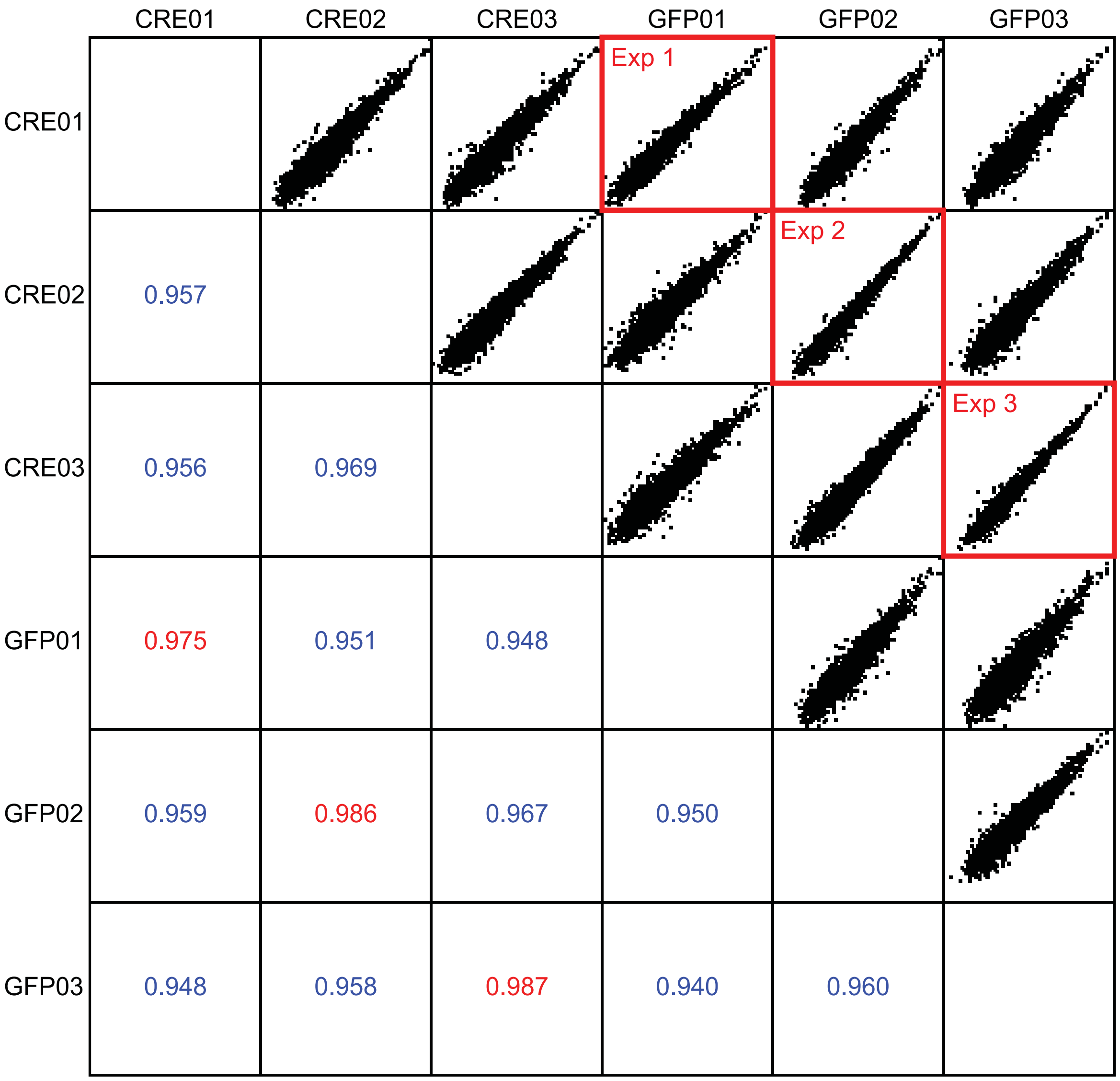

Appendix Figure S1

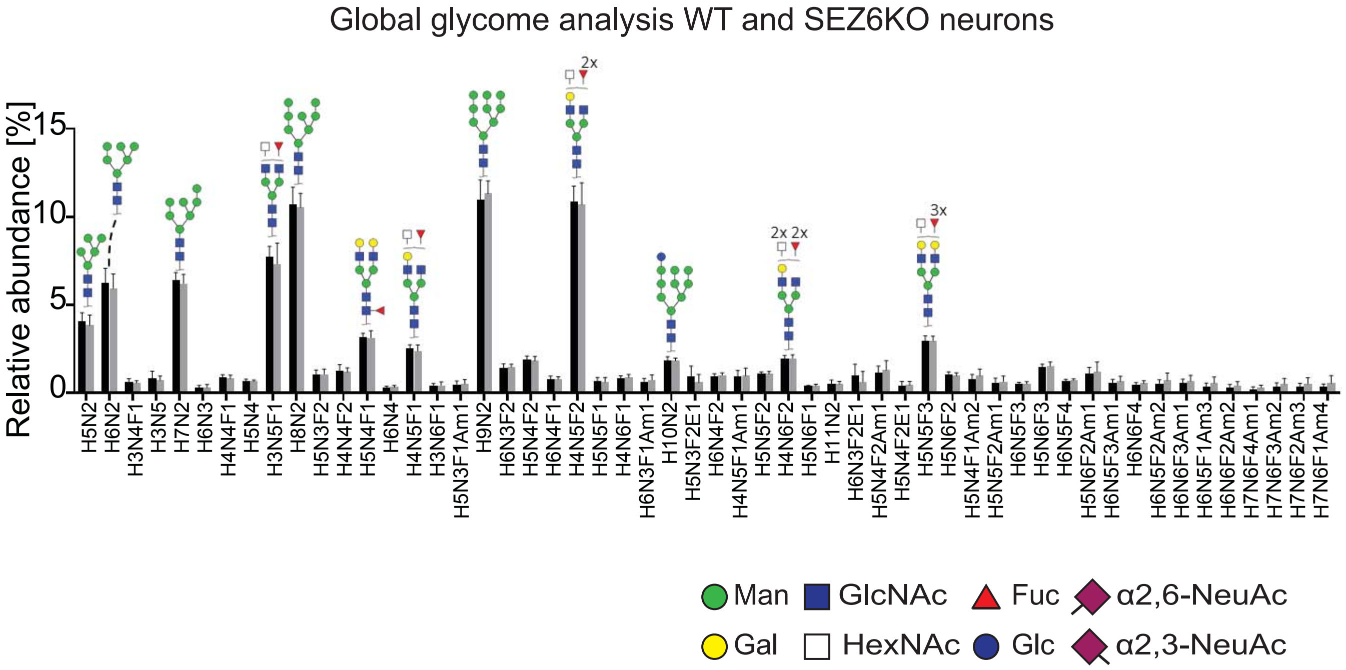

Appendix Figure S2

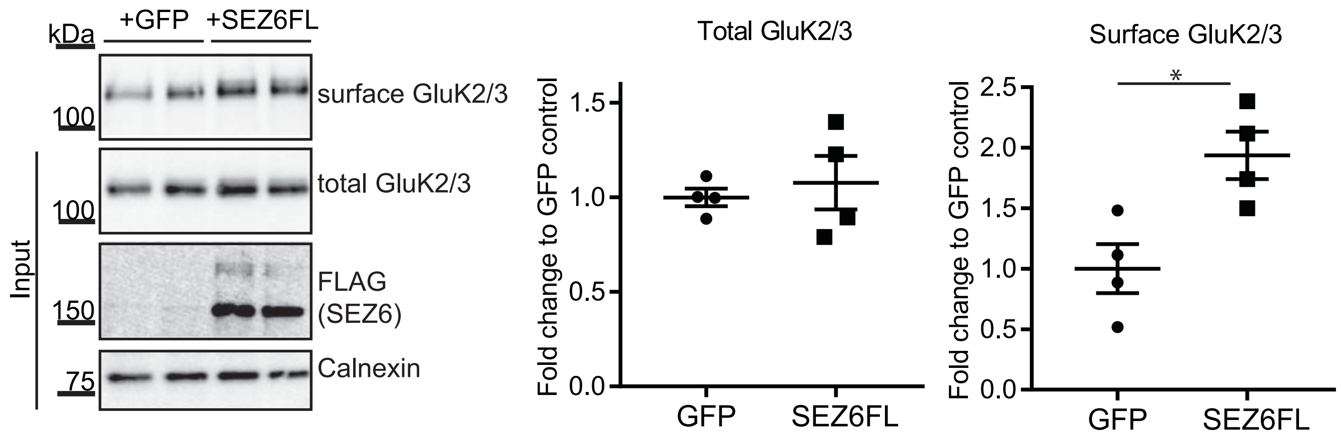

Appendix Figure S3
