# Supplementary material for: Seizure protein 6 controls glycosylation and trafficking of kainate receptor subunits GluK2 and GluK3
Source: EMBO J. 2020 Jun 22;39(15):e103457. doi: 10.15252/embj.2019103457 (PMC7396870; doi:10.15252/embj.2019103457)
Supplement: Supplementary file 2 — Expanded View Figures PDF [file EMBJ-39-e103457-s002.pdf]

Expanded View Figures

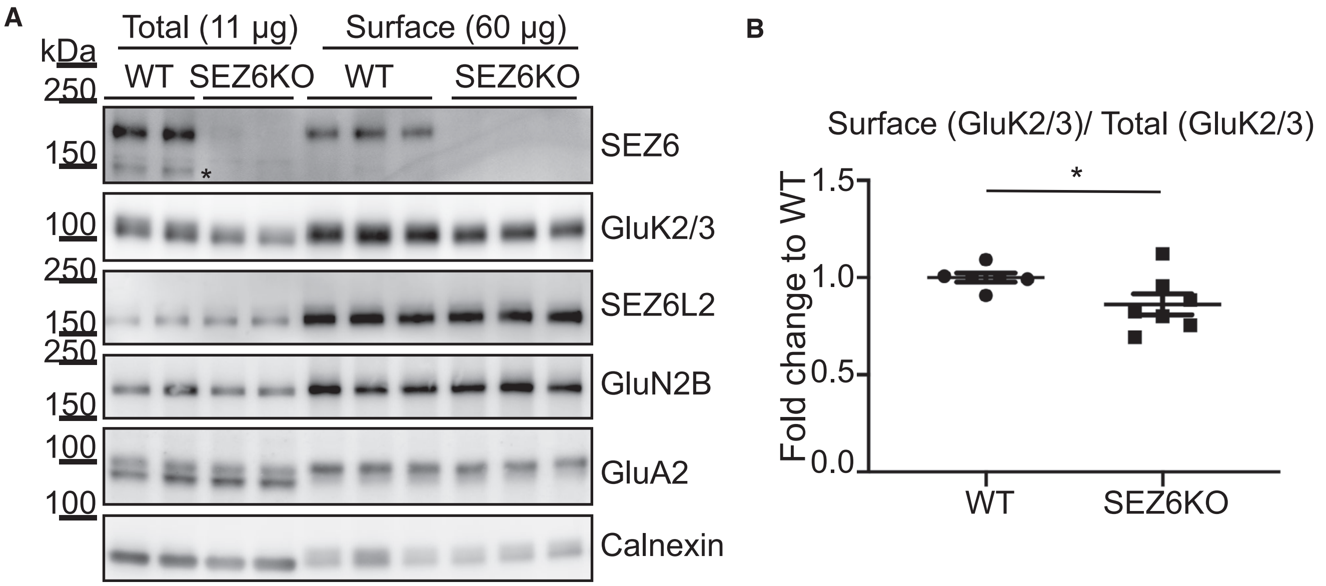

Figure EV1. Quality control of surface enrichment by Sulfo-NHS-Biotin.

A SEZ6KO and WT neurons were biotinylated with Sulfo-NHS-Biotin, and surface proteins were enriched by streptavidin bead pull-down. Total proteins in the lysates (11 µg, "Total") and surface proteins (60 µg, "Surface") were analyzed by immunoblotting. The efficiency of the enrichment is shown by the calnexin depletion, by the absence of immature SEZ6 (black star, Pigoni *et al*, 2016) and by the absence of a second GluA2 band at a lower molecular weight in the surface pull-down compared to the total lysates.

B GluK2/3 was quantified both in total lysates and at the cell surface, and GluK2/3 surface/total ratio was normalized for SEZ6L2 surface/total (plot shows mean ± SEM, 6 WT, and 7 SEZ6KO replicates and Mann–Whitney test were used. Exact **P*-value = 0.047).

Source data are available online for this figure.

Figure EV2. Quantification of impaired GluK2/3 glycosylation.

A Quantification of the WT and KO samples represented in Fig 4A. In the graph, the average intensity along the *y*-axis was plotted. Measurements were done from bottom to top. Thus, the point $x = 0$ marks the bottom of the band. The average of 6 WT and 5 SEZ6KO samples is plotted, with the shadowed area around the curves representing the standard error of the mean (SEM). Analysis of quartiles was done as described in the Materials and Methods section and was performed to focus on the intensity change in the top of the band. Vertical lines in the graph represent the quartiles that divide the area under the curve into 4 equal pieces. Results of the quartile analysis are summarized in the table. Analysis of the fourth quartile (Q4) demonstrates a significant increase in WT vs. SEZ6KO (highlighted lane in the table), consistent with the presence of the uppermost band in the immunoblot under WT but not under SEZ6KO conditions (*t*-test was used, *P*-values reported in the figure).

B In WT brains, GluK2/3 was seen as two closely comigrating bands, whereas the upper one of lower intensity was missing or running even more closely to the lower band of main intensity in SEZ6KO brains (schematic representation on the right). When the brains of SEZ6KO and WT neurons were digested with endoglycosidase H (EndoH), the two closely comigrating bands were converted to two bands of a lower apparent molecular weight, consistent with full deglycosylation of the lowest band and a partial deglycosylation of the upper band (marked in yellow and green in the right panel). In SEZ6KO neurons, the uppermost (green) band was missing and a new band of lower apparent molecular weight was seen that was overlapping with the yellow labeled band and was indicated with the purple asterisk in the SEZ6KO. No difference in the glycosylation of GluA2 was detectable, pointing to a specific effect of SEZ6 on GluK2/3.

C Total levels of GluA2 were quantified in WT and SEZ6 KO neurons and normalized to calnexin (left panel, plot shows mean ± SEM, 3 WT replicates and 3 SEZ6KO replicates were used). Total levels of GluA2 were quantified in WT and SEZ6 KO brains and normalized to calnexin (right panel, plot shows mean ± SEM, 6 WT replicates and 6 SEZ6KO replicates were used).

D SEZ6 was quantified in WT, SEZ6KO, SEZ6LKO, and SEZ6L2KO synaptosomes and normalized for PSD95 levels (plot shows mean ± SEM, 4 replicates per genotype, one-way ANOVA, Dunnett's multiple comparison test vs. WT, ****P*-value < 0.0001).

© 2020 The Authors

A

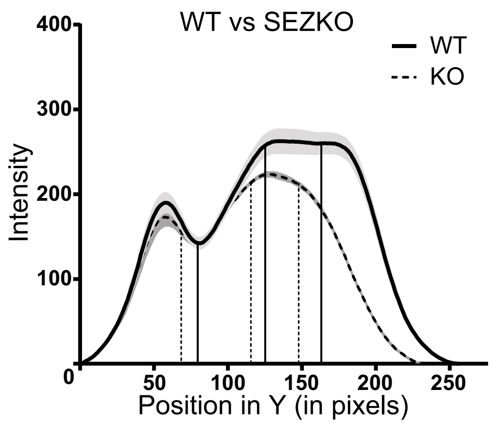

Normalized Average Intensities of Quartiles				
	KO avg. (SEM)	WT avg. (SEM)	WT/KO (SEM)	p-value
Q1	75,31 (2,59)	70,97 (2,30)	0,94 (0,030)	0,240
Q2	140,81 (5,01)	143,39 (5,36)	1,02 (0,038)	0,737
Q3	172,64 (5,50)	169,22 (4,42)	0,98 (0,026)	0,635
Q4	72,98(0,46)	77,70 (1,10)	1,06 (0,015)	0,005

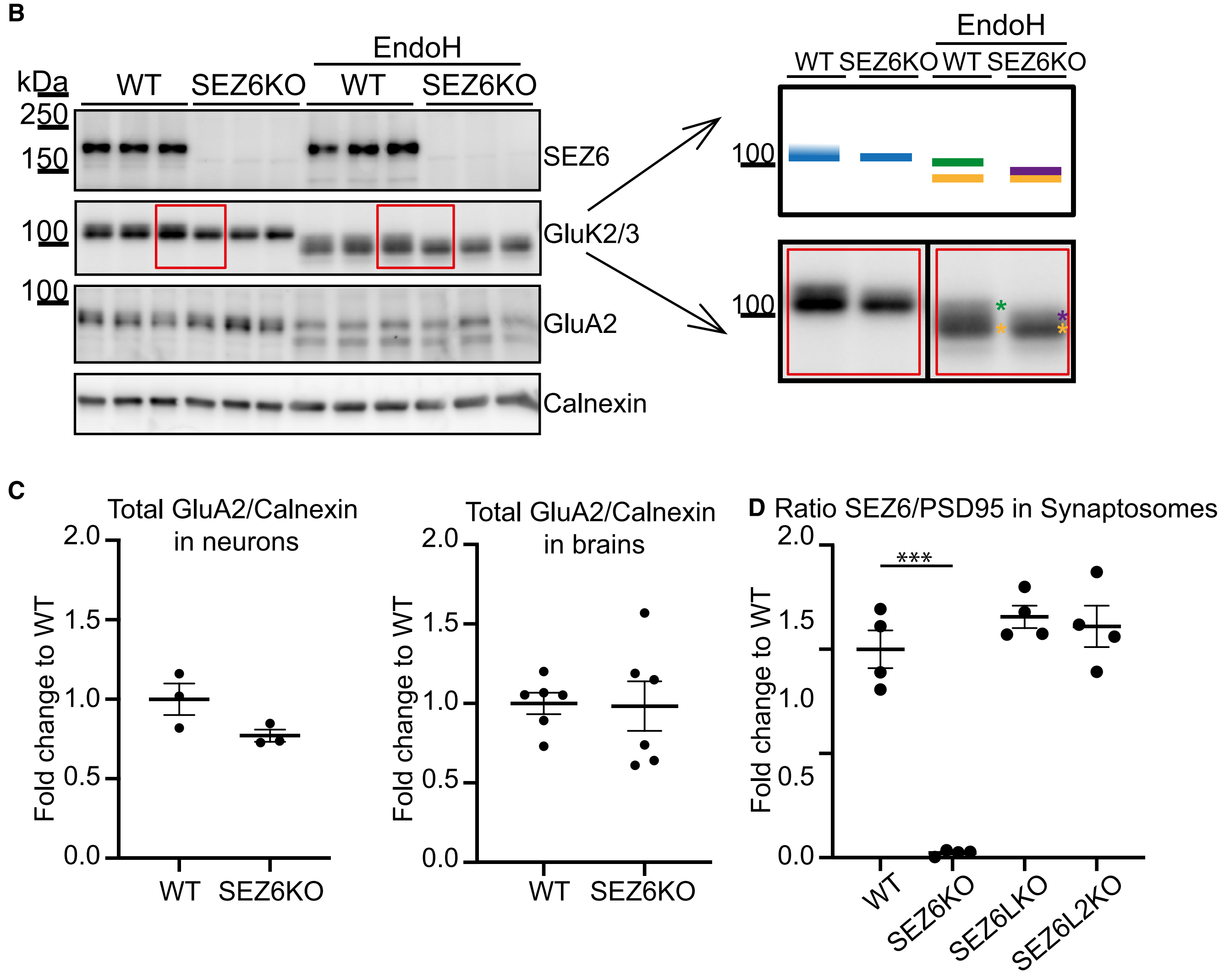

Figure EV2.

 © 2020 The Authors

Figure EV3. SEZ6 and SBP-GFP-GluK2 colocalizes in TGN-derived vesicles.

A At timepoint "0 min", SBP-GFP-GluK2 colocalizes with ER marker (anti-calnexin(mouse) and anti-mouse-Alexa594). At "20 min", SBP-GFP-GluK2 colocalizes with Golgi markers GM130 (mouse and anti-mouse-Alexa594) and TGN46 (sheep and anti-sheep-Alexa594) and is also found in some vesicles (white arrowheads). At "40 min", SBP-GFP-GluK2 vesicles partially colocalize with SBP-LyzC-RFP vesicles (white arrowheads point to Golgi-derived vesicles containing SBP-GFP-GluK2 and SBP-LyzC-RFP). Size bars represent 5 μm.

B RUSH experiment was performed co-transfecting SBP-mCherry-GluK2 in HEK293T with the control plasmid (inactive Cre) or with SEZ6FL construct. The number of vesicles containing SBP-mCherry-GluK2 was determined at 20 and 40 min after biotin treatment. When SEZ6FL was co-transfected, the number of vesicles containing SBP-mCherry-GluK2 was significantly higher compared to the control condition (plot shows mean ± S.D., 3 independent experiments, Mann–Whitney test was used to compare SEZ6FL and control at each time point. At 20 min, there was no significant difference, at 40 min ****P-value < 0.0001).

C Colocalization of TGN-derived vesicles of SBP-GFP-GluK2 in HEK293T cells co-transfected with SEZ6ΔcytoER-HA, SEZ6FL-HA, and SBP-LyzC-RFP at "40 min" timepoint of RUSH experiment. White arrowheads point to vesicles containing SBP-GFP-GluK2 colocalizing with SEZ6FL-HA or SBP-LyzC-RFP, respectively. Size bars represent 5 μm.

D Scatter dot plot represents percentage of colocalizing vesicles divided by total number of SBP-GFP-GluK2 vesicles. Plot shows mean and SD. Vesicle counts from 10 to 15 cells from 2 independent experiments are shown. Analysis was performed with non-parametric Kruskal–Wallis test ****P-value < 0.0001, followed by Dunn's multiple comparisons test SEZ6ΔcytoER-HA vs. SEZ6FL-HA **P-value $= 0.0093$, SEZ6ΔcytoER-HA vs. SBP-LyzC-RFP ****P-value < 0.0001, SEZ6FL-HA vs. SBP-LyzC-RFP *P-value $= 0.0241$.

© 2020 The Authors

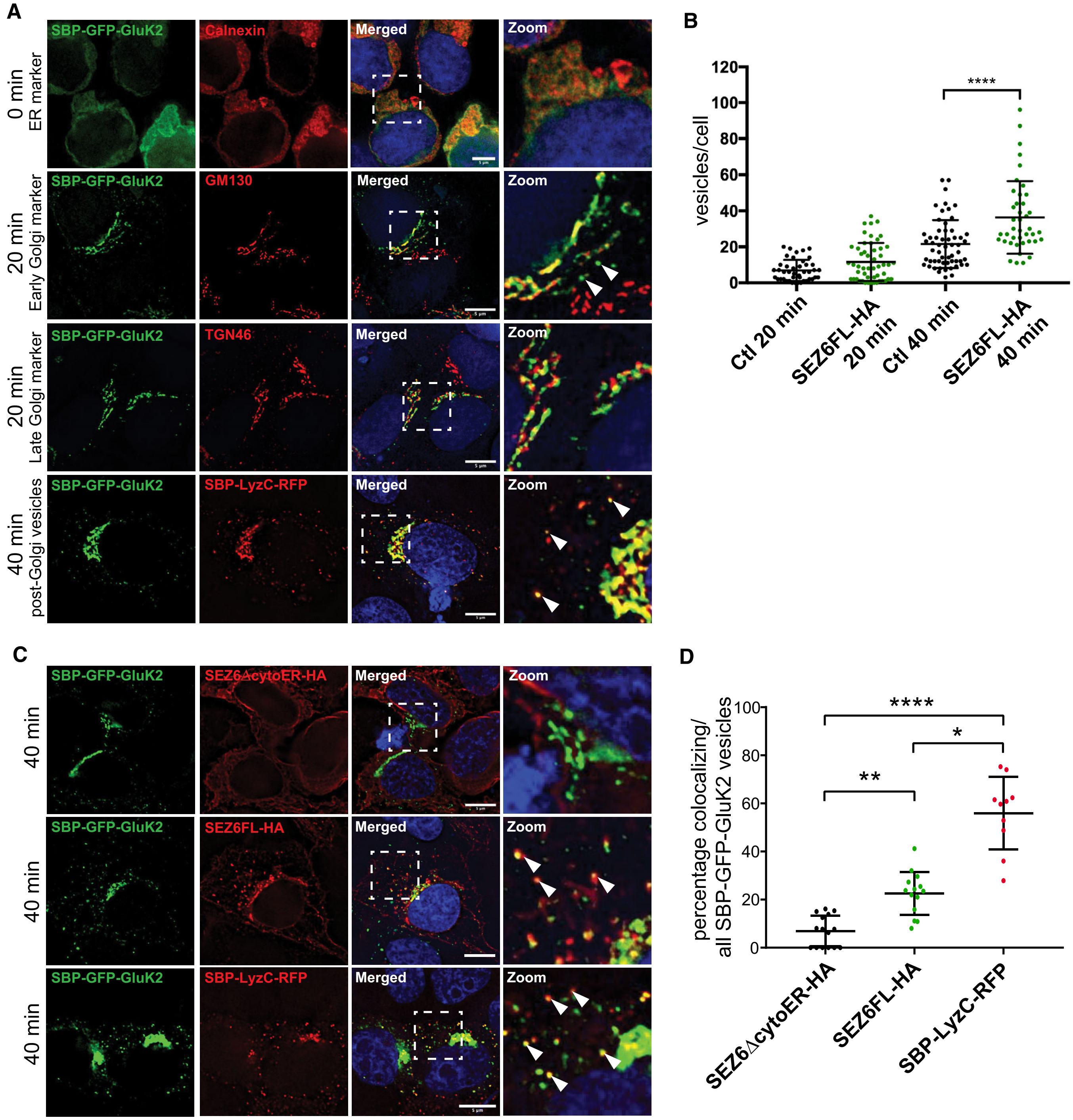

Figure EV3.

 © 2020 The Authors

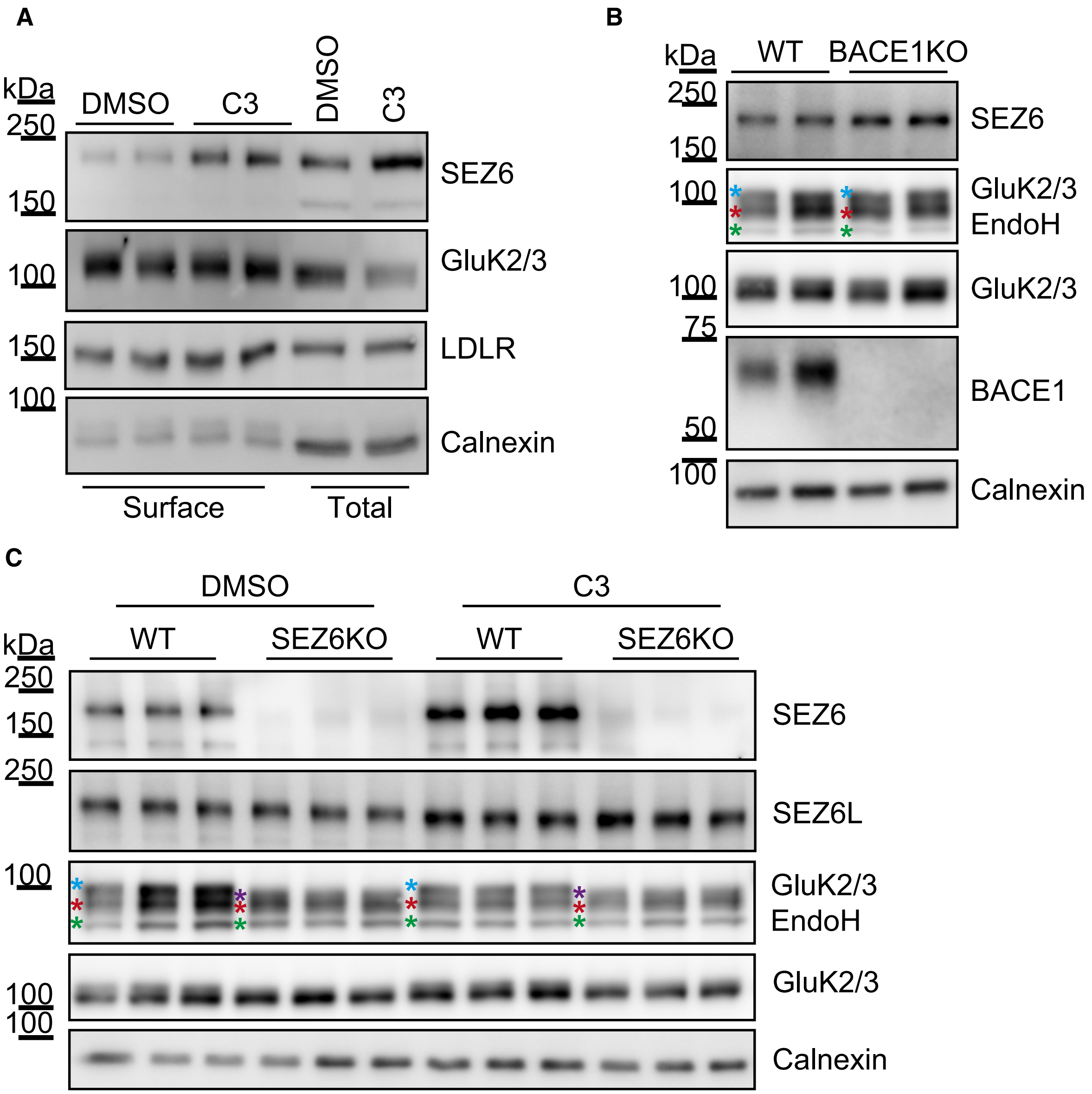

Figure EV4. BACE1 cleavage does not affect the function of SEZ6 as GluK2/3 regulator.

A WT neurons were treated with the BACE inhibitor C3 or DMSO as control and surface biotinylation with Sulfo-NHS-Biotin was performed. BACE1 inhibition did not only prevent SEZ6ecto formation (Pigoni *et al*, 2016), but also increased full-length SEZ6 levels at the cell surface compared to the control condition. Even though SEZ6 full length accumulated on the cell surface of BACE-inhibitor-treated neurons, no change in GluK2/3 amounts at the cell surface was detected.

B Membrane fraction of WT and BACE1KO brains was digested with EndoH. As expected, SEZ6 full length accumulates in the membrane fraction of BACE1KO brains. No change in GluK2/3 glycosylation or total amount was detected. Color coding for asterisks is the same as in Fig 4A.

C WT and SEZ6KO neurons were treated with the BACE1 inhibitor C3 or DMSO as control and total lysates were analyzed after EndoH digestion. Even though SEZ6 full length accumulates upon BACE inhibition, and GluK2/3 presents immature glycosylation in the SEZ6KO neurons, no change of GluK2/3 glycosylation or total amounts was seen when WT neurons were treated with C3. This indicates that both surface localization (A) and glycosylation (B and C) of GluK2/3 are likely to have reached their maximum in WT cells and cannot be enhanced by increased SEZ6 levels, as induced here through BACE1 inhibition. Color coding for asterisks is the same as in Fig 4A.

Source data are available online for this figure.

© 2020 The Authors

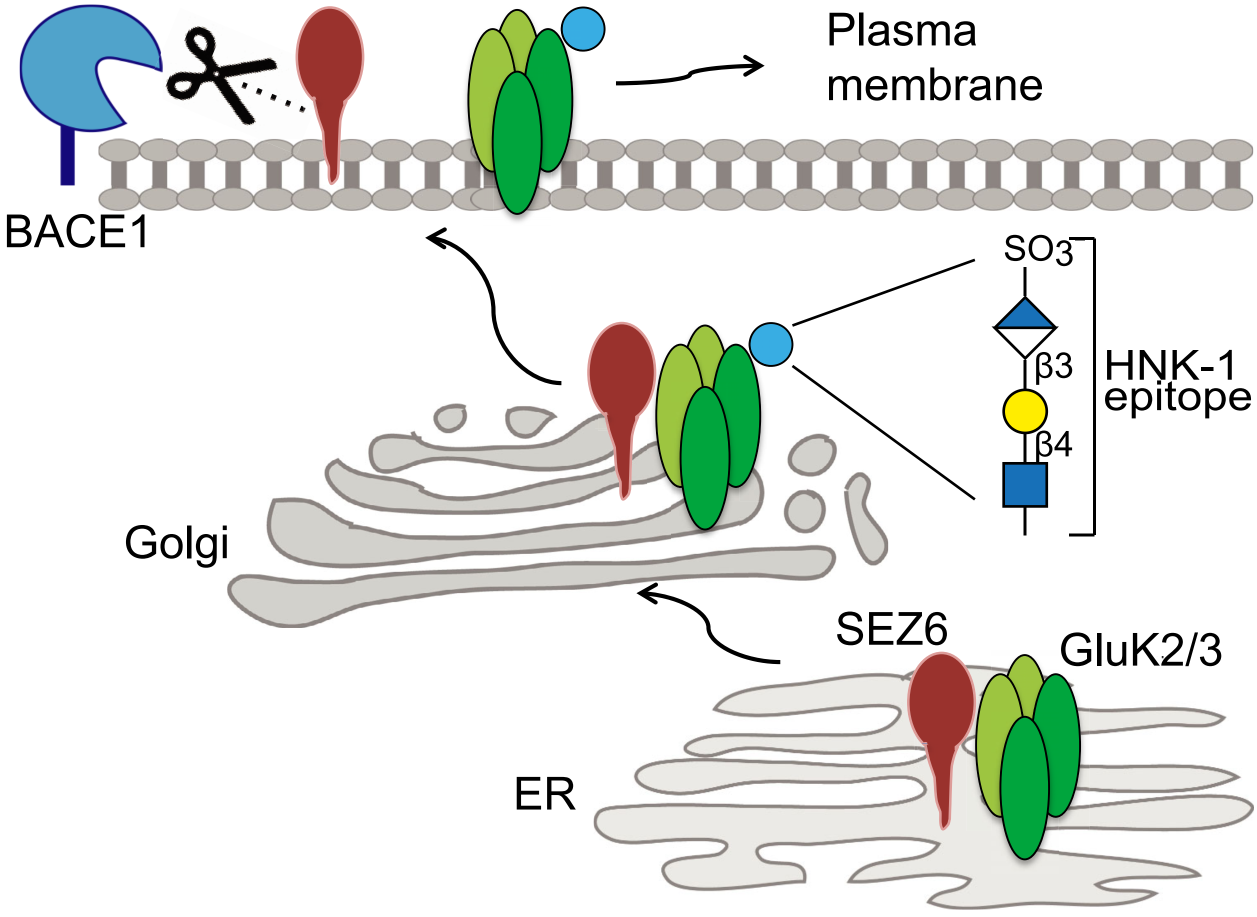

Figure EV5. Proposed model for GluK2/3 regulation mediated by SEZ6.

SEZ6 interacts with GluK2 in the early secretory pathway (ER) and facilitates its trafficking through the secretory pathway. While trafficking into the Golgi, GluK2/3 undergoes several sugar modifications, including HNK-1 modification. Once reached the cell surface, SEZ6 and GluK2/3 separate and become independent from each other, explaining why BACE1 cleavage does not affect GluK2/3 maturation. When SEZ6 is not present, GluK2/3 trafficking is impaired and also its function at the cell surface.

 © 2020 The Authors
