# Supplementary material for: Seizure protein 6 controls glycosylation and trafficking of kainate receptor subunits GluK2 and GluK3
Source: EMBO J. 2020 Jun 22;39(15):e103457. doi: 10.15252/embj.2019103457 (PMC7396870; doi:10.15252/embj.2019103457)
Supplement: Supplementary file 3 — Table EV1 [file EMBJ-39-e103457-s003.docx]

**Table EV1**

**Proteins significantly changed on the cell surface of SEZ6KO neurons compared to WT.** Proteins with LFQ intensity lower than log2 ratio (SEZ6KO/WT)=-0.5 (0.71 fold change) or higher than log2 ratio(SEZ6KO/WT)=0.5 (1.4 fold change) and a p-value lower than 0.05 were considered as hits. Proteins are sorted according to their fold change (“Ratio”) and their p-values is reported (“p-value”). Membrane (“Membrane”), soluble (“Soluble”) and proteins with unknown classification (“Unknown”) were detected, according to Uniprot.

| **Protein IDs** | **Gene names** | **Protein names** | **Ratio** | **p-value** | **Protein**  **type** |
| --- | --- | --- | --- | --- | --- |
| B1AS29 | Grik3/  GluK3 | Glutamate receptor ionotropic. kainate 3 | 0.43 | 0.02 | Membrane |
| D3YTM0 | Cers2 | Ceramide synthase 2 | 0.45 | 0.01 | Membrane |
| Q9ER00 | Stx12 | Syntaxin-12 | 0.46 | 0.05 | Membrane |
| P39087 | Grik2/  GluK2 | Glutamate receptor ionotropic. kainate 2 | 0.52 | 0.01 | Membrane |
| P61089 | Ube2n | Ubiquitin-conjugating enzyme E2 N | 0.54 | 0.03 | Soluble |
| O54865 | Gucy1b3 | Guanylate cyclase soluble subunit beta-1 | 0.57 | 0.05 | Soluble |
| Q8BTX9 | Hsdl1 | Inactive hydroxysteroid dehydrogenase-like protein 1 | 0.58 | 0.03 | Soluble |
| O08915 | Aip | AH receptor-interacting protein | 0.59 | 0.01 | Soluble |
| P11627 | L1cam | Neural cell adhesion molecule L1 | 0.62 | 0.02 | Membrane |
| Q9Z0P4-2 | Palm | Paralemmin-1 | 0.63 | 0.01 | Membrane |
| P97785-2 | Gfra1 | GDNF family receptor alpha-1 | 0.68 | 0.04 | Membrane |
| Q8BYI8 | Kiaa1467 | Uncharacterized protein KIAA1467 | 0.70 | 0.01 | Membrane |
| O08912 | Galnt1 | Polypeptide N-acetylgalactosaminyltransferase 1 | 0.70 | 0.03 | Membrane |
| Q9R1R8 | Rdh11 | Retinol dehydrogenase 11 | 0.71 | 0.03 | Unknown |

| **Protein IDs** | **Gene names** | **Protein names** | **Ratio** | **p-value** | **Protein**  **Type** |
| --- | --- | --- | --- | --- | --- |
| Q99LD9 | Eif2b2 | Translation initiation factor eIF-2B subunit beta | 2.00 | 0.01 | Unknown |
| Q9CWR2 | Smyd3 | Histone-lysine N-methyltransferase SMYD3 | 1.68 | 0.04 | Soluble |
| Q9D6R2 | Idh3a | Isocitrate dehydrogenase [NAD] subunit alpha. mitochondrial | 1.66 | 0.05 | Soluble |
| Q8BUY9 | Pggt1b | Geranylgeranyl transferase type-1 subunit beta | 1.64 | 0.04 | Unknown |
| Q8R3I2-3 | Mboat2 | Lysophospholipid acyltransferase 2 | 1.64 | 0.01 | Membrane |
| O88986 | Gcat | 2-amino-3-ketobutyrate coenzyme A ligase. mitochondrial | 1.64 | 0.05 | Soluble |
| F6S1R2 | Ahsa2 | Activator of 90 kDa heat shock protein ATPase homolog 2 | 1.62 | 0.02 | Unknown |
| Q8R323 | Rfc3 | Replication factor C subunit 3 | 1.45 | 0.05 | Soluble |
| Q8K2M0-2 | Mrpl38 | 39S ribosomal protein L38. mitochondrial | 1.44 | 0.04 | Soluble |
