# Supplementary material for: Seizure protein 6 controls glycosylation and trafficking of kainate receptor subunits GluK2 and GluK3
Source: EMBO J. 2020 Jun 22;39(15):e103457. doi: 10.15252/embj.2019103457 (PMC7396870; doi:10.15252/embj.2019103457)
Supplement: Supplementary file 4 — Table EV2 [file EMBJ-39-e103457-s004.docx]

**Table EV2**

**Proteins co-immunoprecipitated with GluK2/3 in endogenous conditions.** GluK2/3 and SEZ6 as control were immunoprecipitated from WT brain homogenates (n=6). Proteins co-immunoprecipitated with GluK2/3 in more than 4 out of 6 replicates and detected in less than 2 out of 6 controls, are reported in the table. Proteins are sorted according to their average LFQ intensity, showing at the top of the table the proteins giving a stronger signal. Neto2 was co-immunoprecipitated with GluK2/3 in endogenous conditions, but SEZ6 was not detectable (“NaN”).

| **Protein IDs** | **Gene names** | **Protein names** | **Average LFQ intensity^#^** |
| --- | --- | --- | --- |
| B1AS29 | Grik3/ GluK3 | Glutamate receptor ionotropic, kainate 3 | 31,97 |
| P39087 | Grik2/ GluK2 | Glutamate receptor ionotropic, kainate 2 | 31,64 |
| Q61626 | Grik5/  GluK5 | Glutamate receptor ionotropic, kainate 5 | 28,25 |
| P47757-2 | Capzb | F-actin-capping protein subunit beta | 25,30 |
| E9Q171 | Nfasc | Neurofascin | 25,23 |
| Q8BMF5 | Grik4 | Glutamate receptor ionotropic, kainate 4 | 24,79 |
| O09061 | Psmb1 | Proteasome subunit beta type-1 | 24,57 |
| P47708 | Rph3a | Rabphilin-3A | 24,05 |
| Q60692 | Psmb6 | Proteasome subunit beta type-6 | 23,95 |
| Q3U2G2 | Hspa4 | Heat shock 70 kDa protein 4 | 23,87 |
| Q8BNJ6 | Neto2 | Neuropilin and tolloid-like protein 2 | 23,76 |
| P35486 | Pdha1 | Pyruvate dehydrogenase E1 component subunit alpha, somatic form, mitochondrial | 23,73 |
| P59016 | Vps33b | Vacuolar protein sorting-associated protein 33B | 22,12 |
| Q7TSK2 | Sez6 | Seizure protein 6 | NaN |

^#^log2 scale
