# Supplementary figures and images for: Seizure protein 6 controls glycosylation and trafficking of kainate receptor subunits GluK2 and GluK3
Source: EMBO J. 2020 Jun 22;39(15):e103457. doi: 10.15252/embj.2019103457 (PMC7396870; doi:10.15252/embj.2019103457)

Figure EV1

A

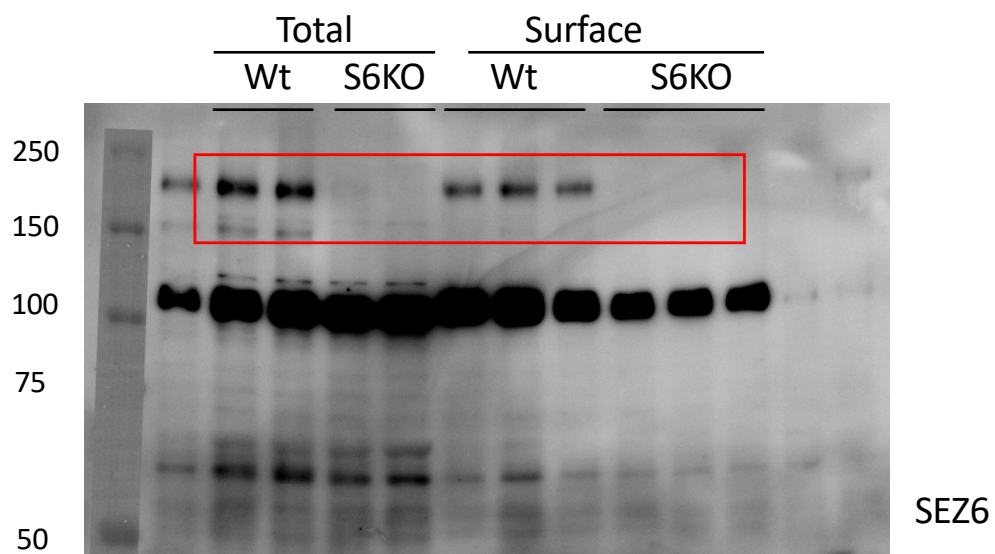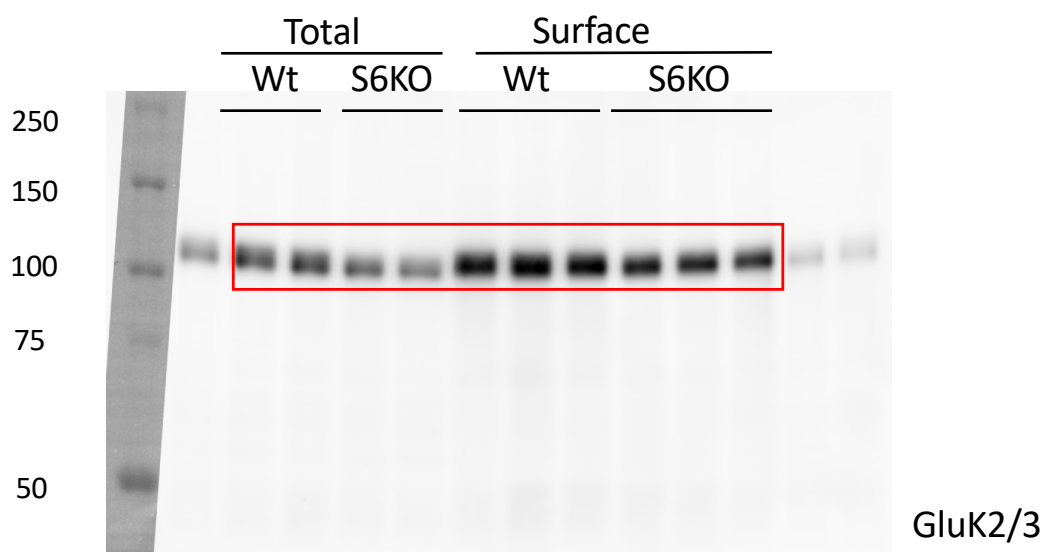

Supplement: Supplementary file 6 — Source Data for Expanded View and Appendix [file EMBJ-39-e103457-s010.zip › EV_and_Appendix_Source_Data/EMBOJ-2019-103457R1-Figure_EV1_Source_Data-sd.pdf]

## Appendix Figure S3

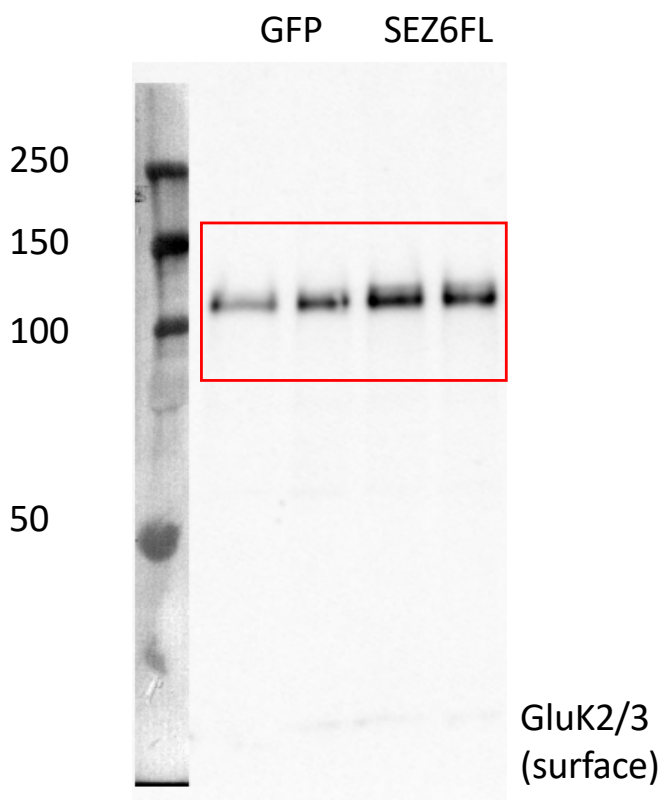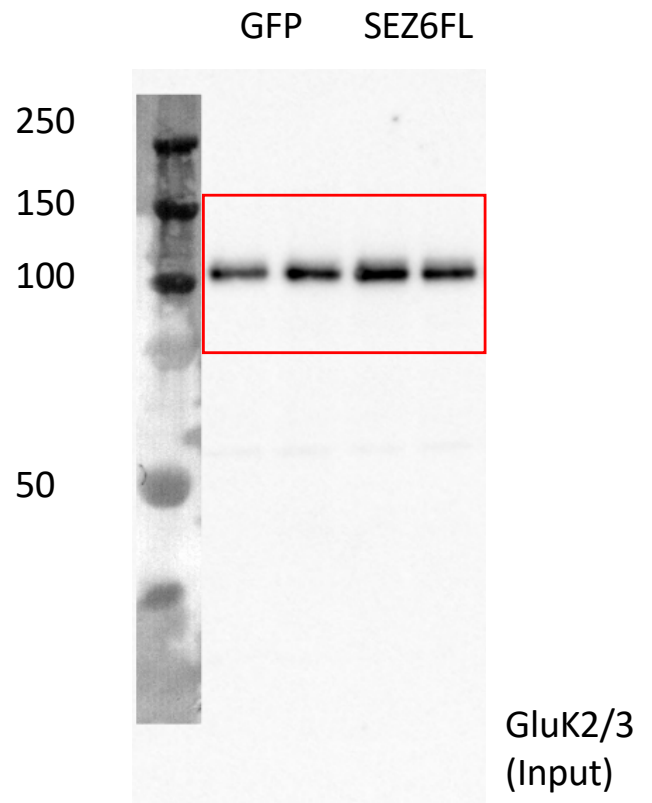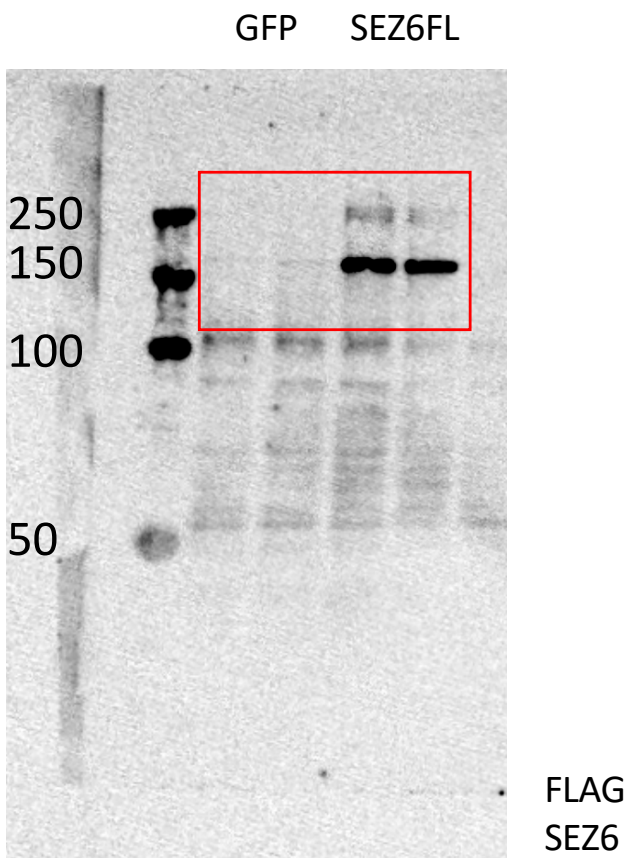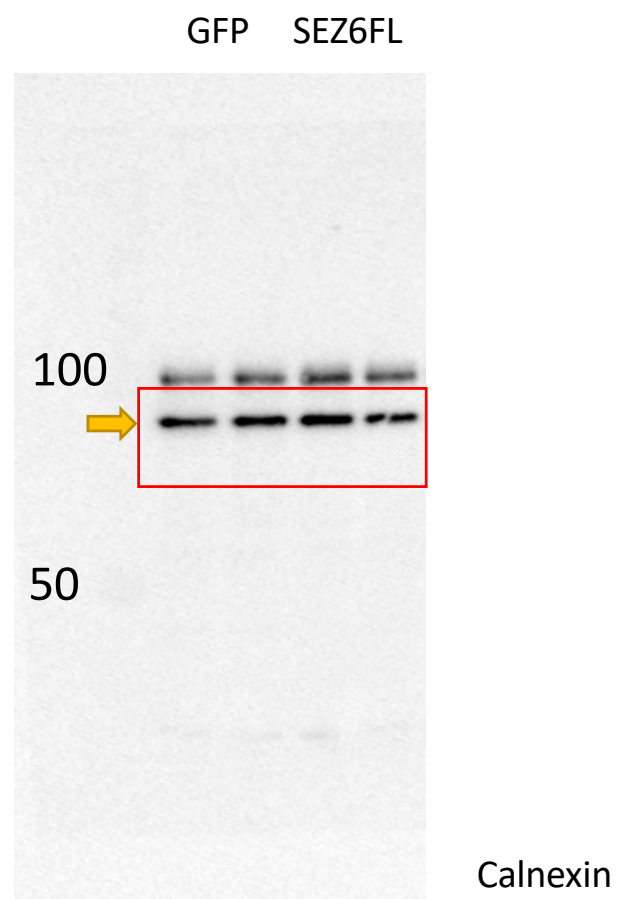

Supplement: Supplementary file 6 — Source Data for Expanded View and Appendix [file EMBJ-39-e103457-s010.zip › EV_and_Appendix_Source_Data/EMBOJ-2019-103457R1-Figure_S3_Appendix_Source_Data-sd.pdf]

Figure EV4

# A

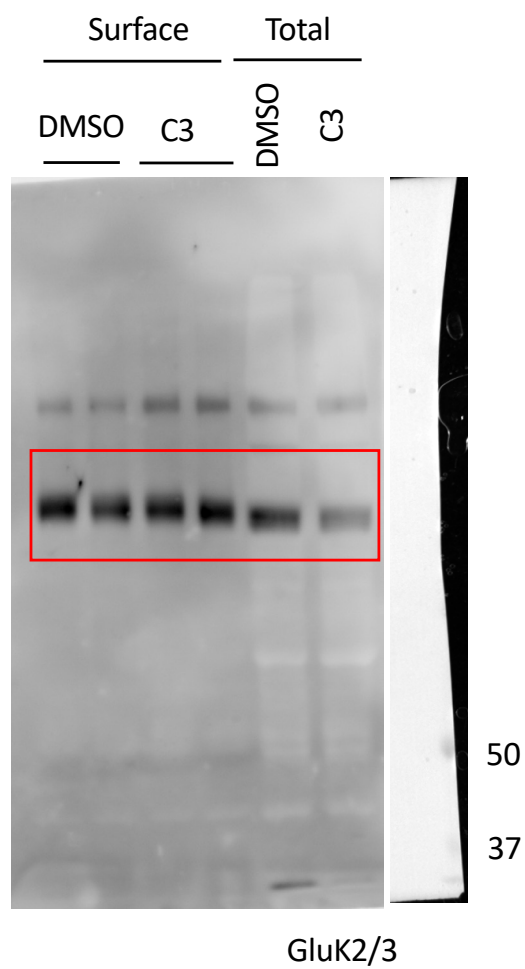

# B

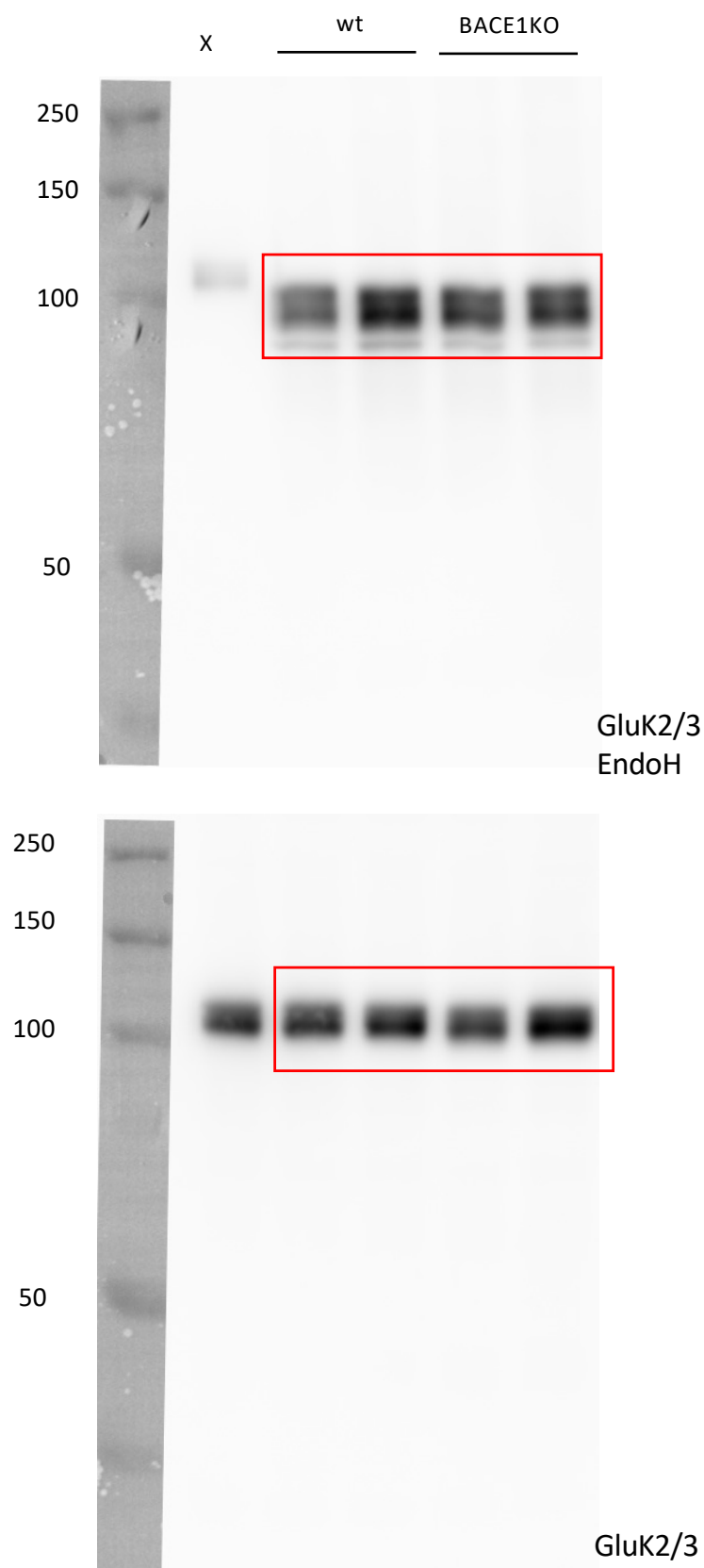

C

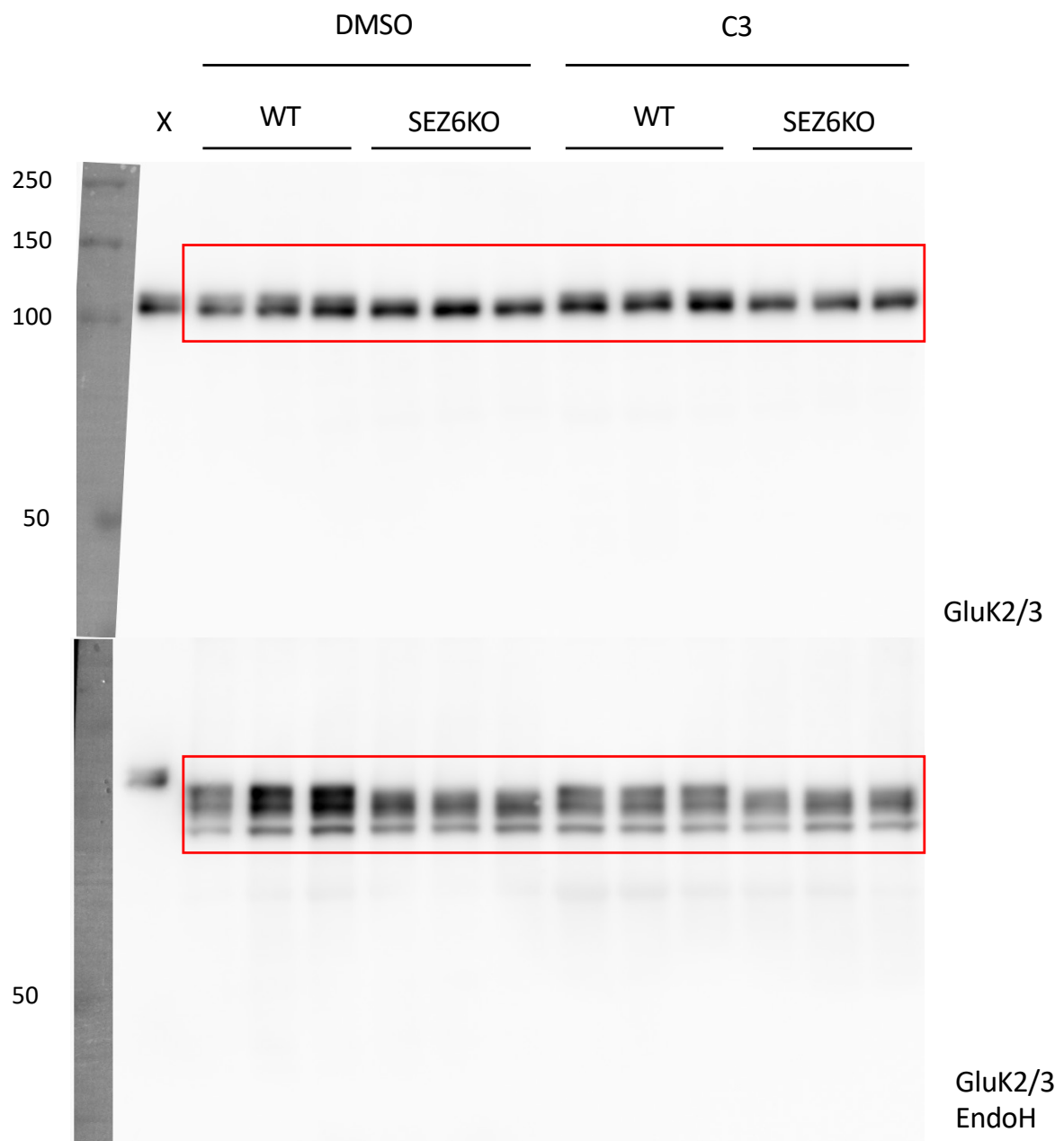

Supplement: Supplementary file 6 — Source Data for Expanded View and Appendix [file EMBJ-39-e103457-s010.zip › EV_and_Appendix_Source_Data/EMBOJ-2019-103457R1-Figure_EV4_Source_Data-sd.pdf]

Figure 2

A

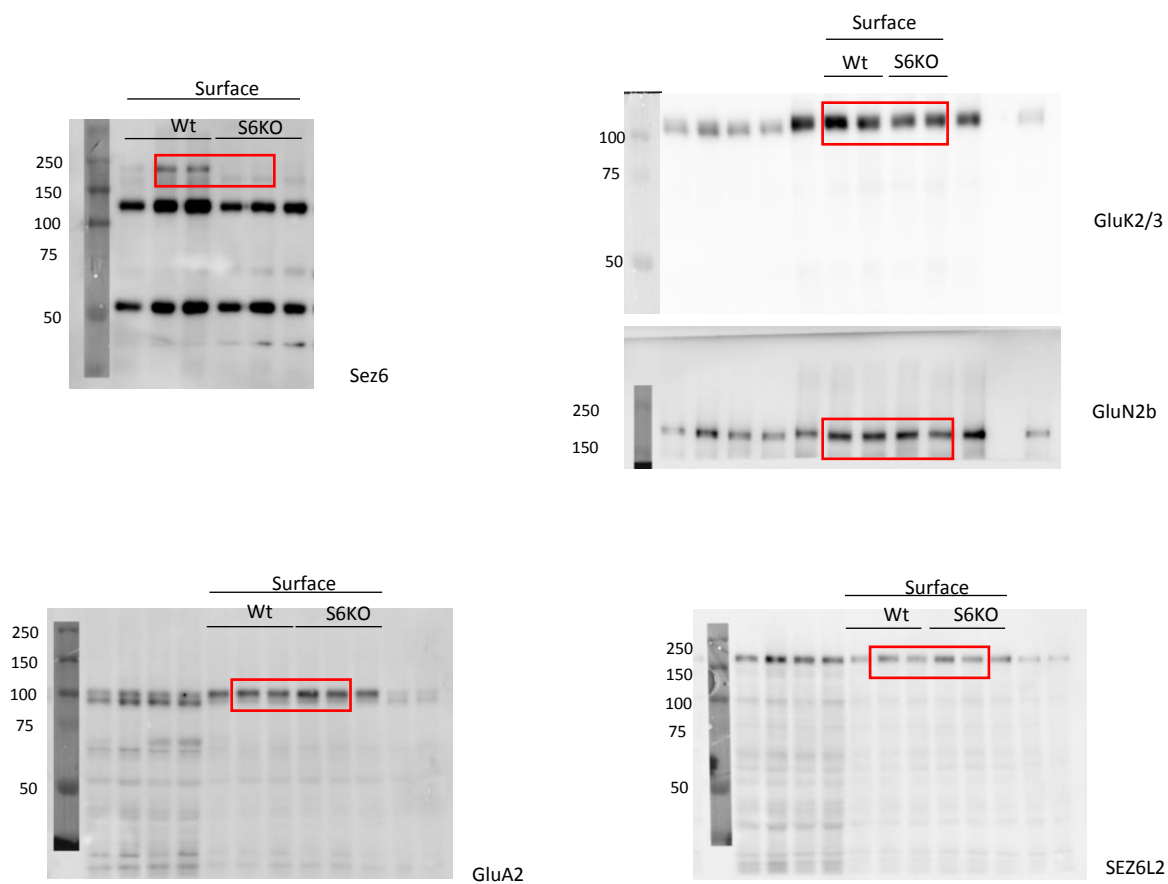

Supplement: Supplementary file 8 — Source Data for Figure 2 [file EMBJ-39-e103457-s006.pdf]

Figure 4

A

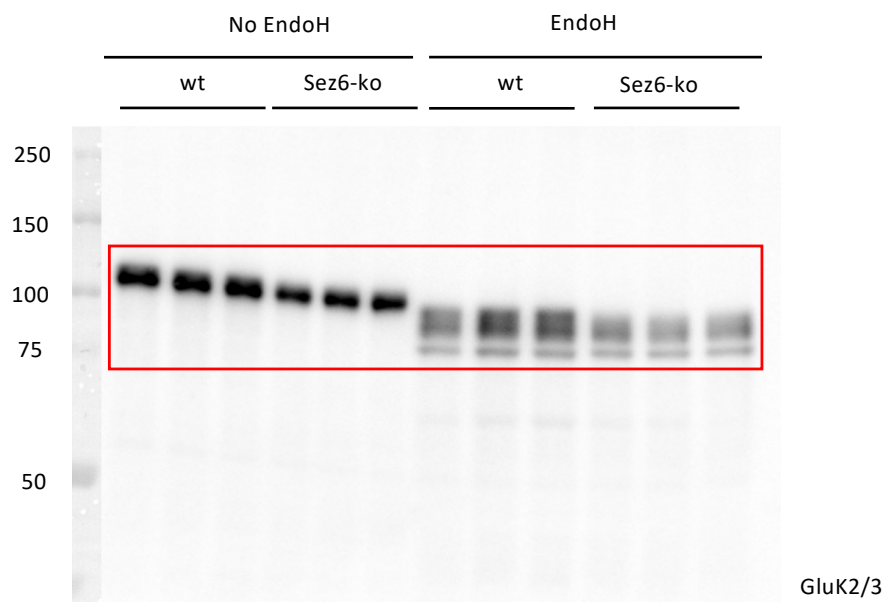

B

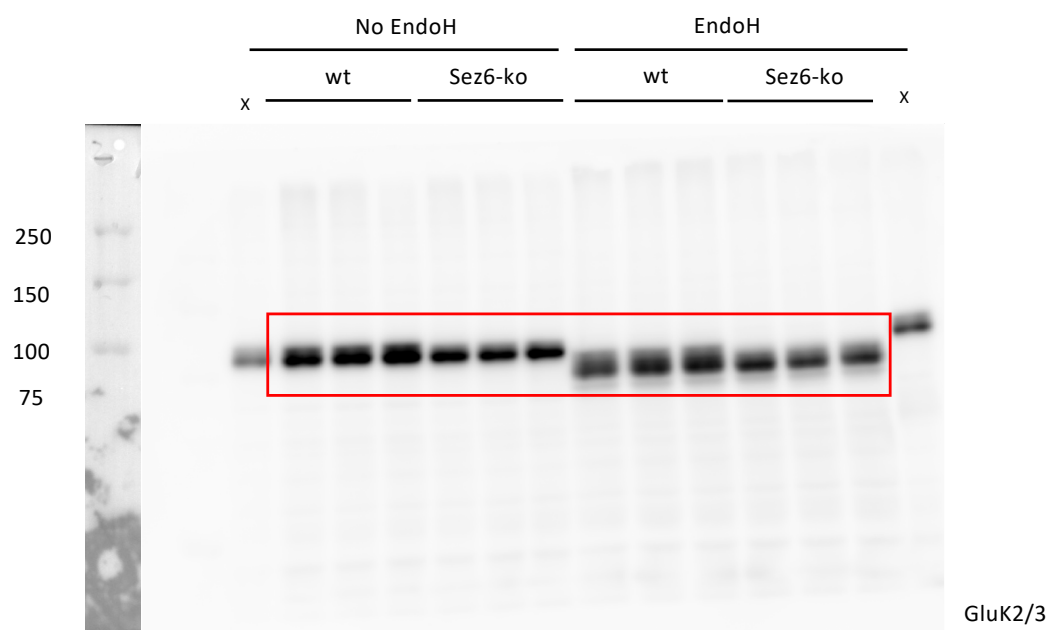

E

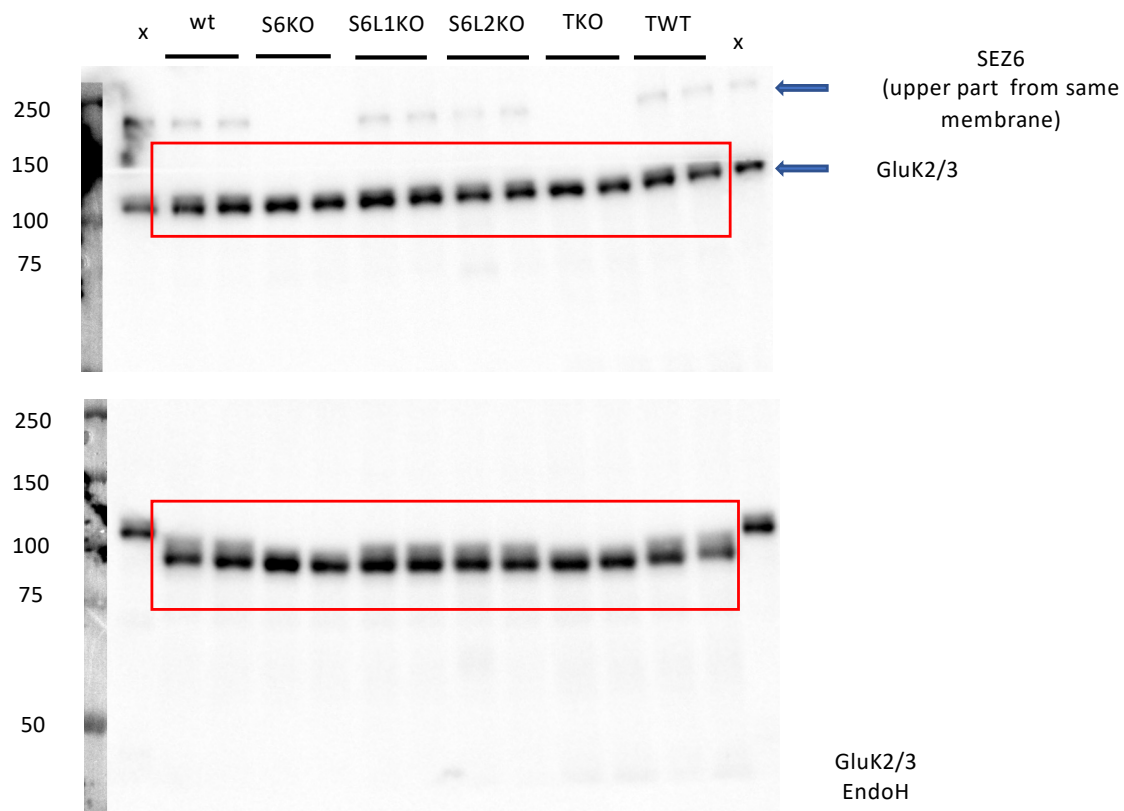

F

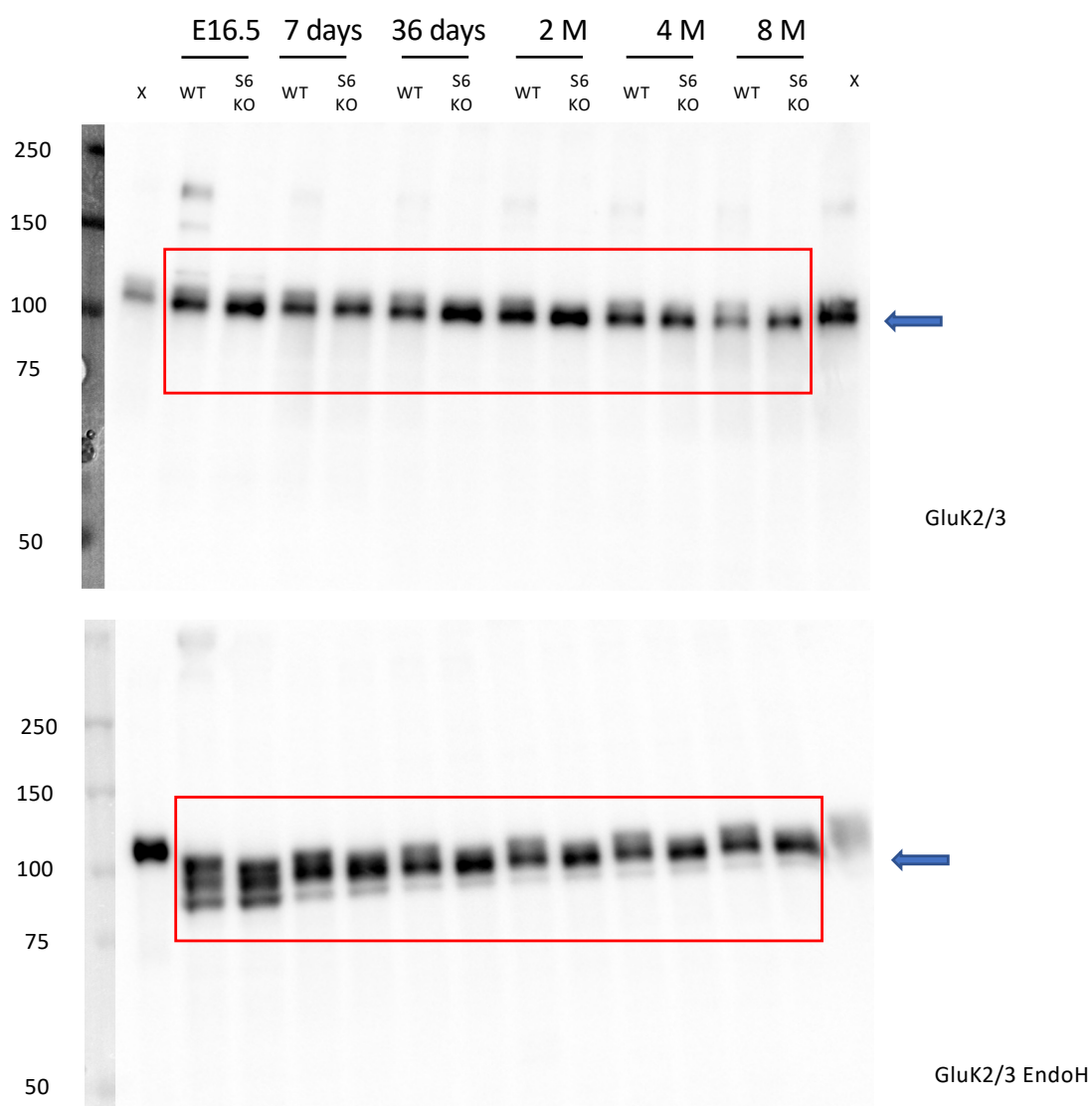

Supplement: Supplementary file 9 — Source Data for Figure 4 [file EMBJ-39-e103457-s007.pdf]

Figure 5

# B

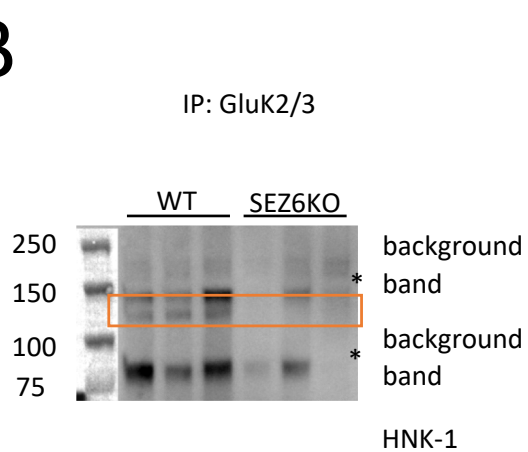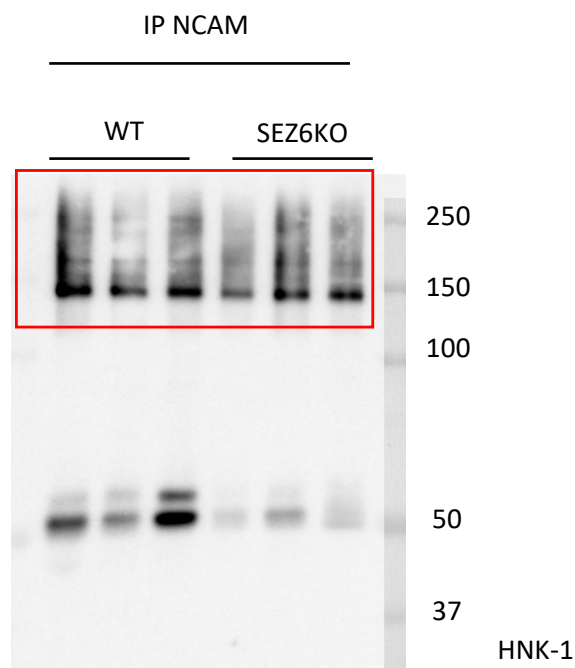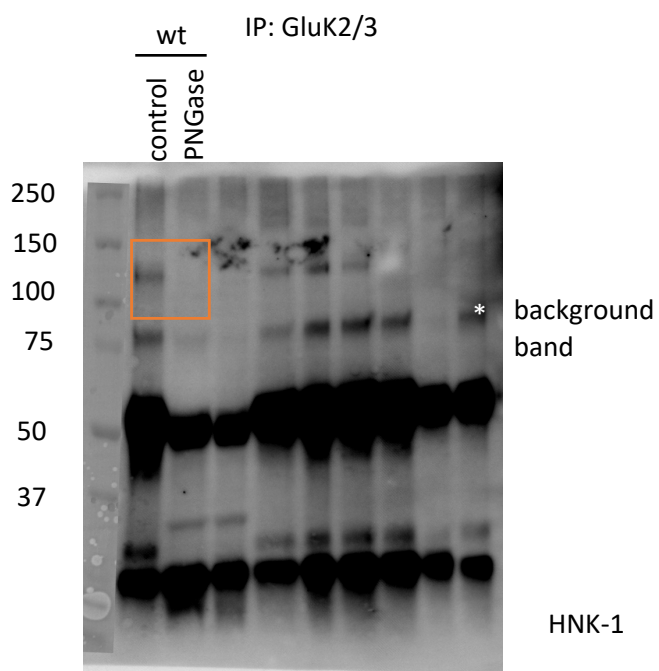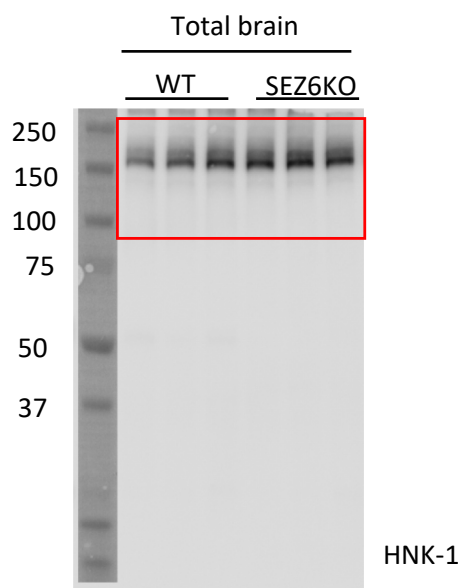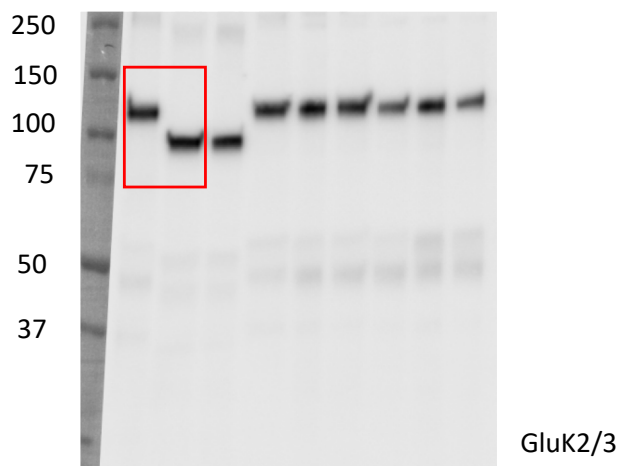

Supplement: Supplementary file 10 — Source Data for Figure 5 [file EMBJ-39-e103457-s008.pdf]
