# Supplementary material for: Seizure protein 6 controls glycosylation and trafficking of kainate receptor subunits GluK2 and GluK3
Source: EMBO J. 2020 Jun 22;39(15):e103457. doi: 10.15252/embj.2019103457 (PMC7396870; doi:10.15252/embj.2019103457)

Figure 8

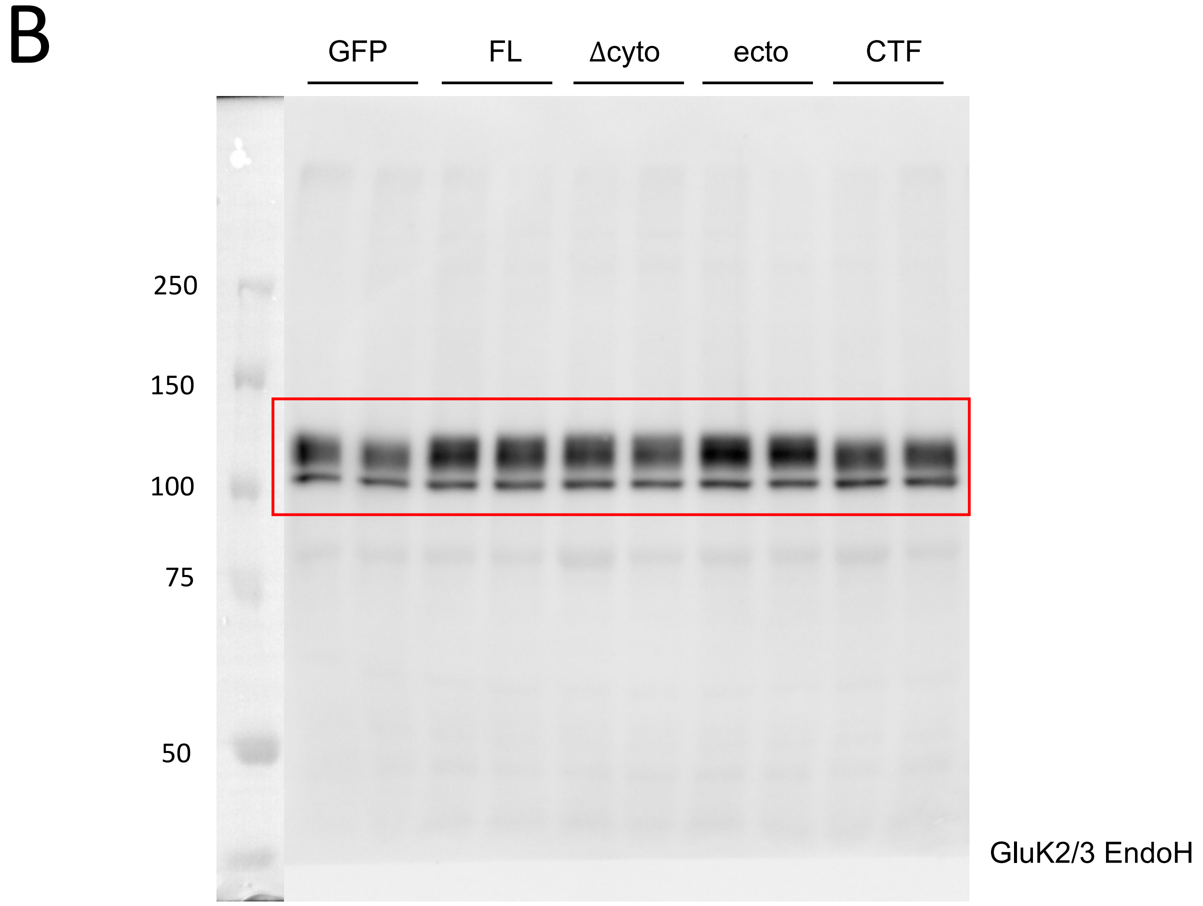
B
GFP
FL
Δcyto
ecto
CTF
250
150
100
75
50
GluK2/3 EndoH

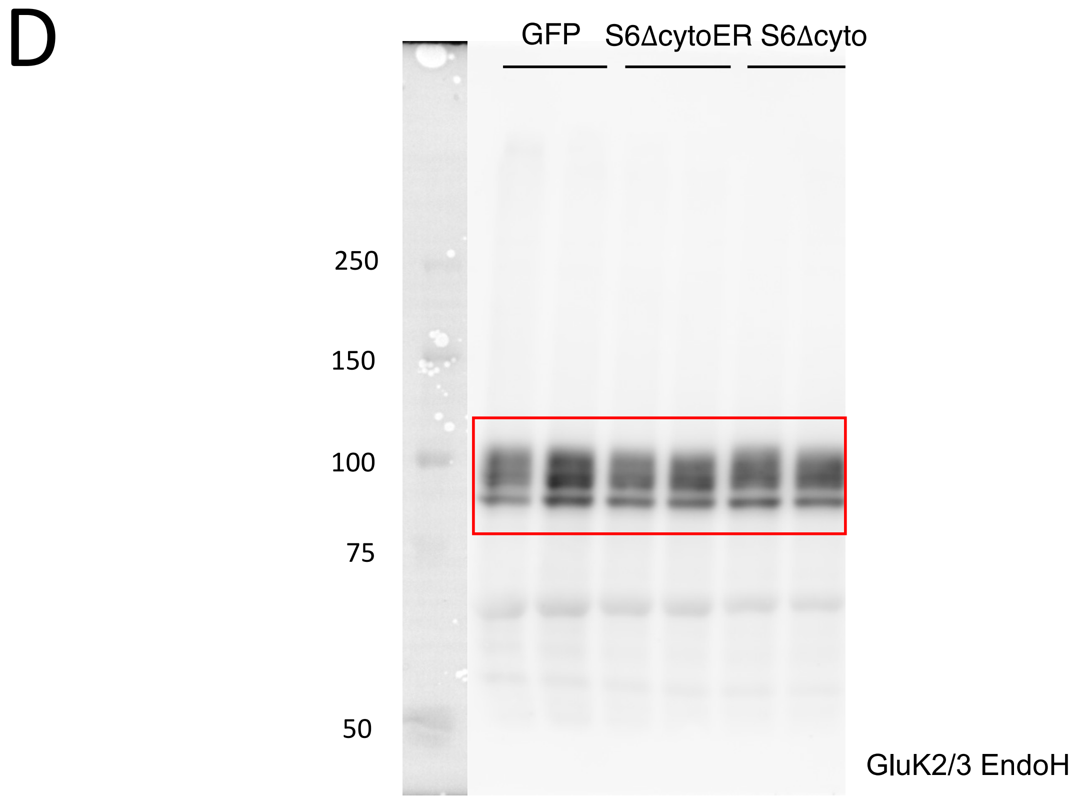
D
GFP
S6ΔcytoER
S6Δcyto
250
150
100
75
50
GluK2/3 EndoH

E

Precipitation with
GluK2/3:
1 no transfection
2 GluK+GFP
3-4 GluK+SEZ6FL
5-6 GluK+SEZ6deltacyto
7-8 GluK+SEZ6ecto
9-10 GluK+SEZ6CTF

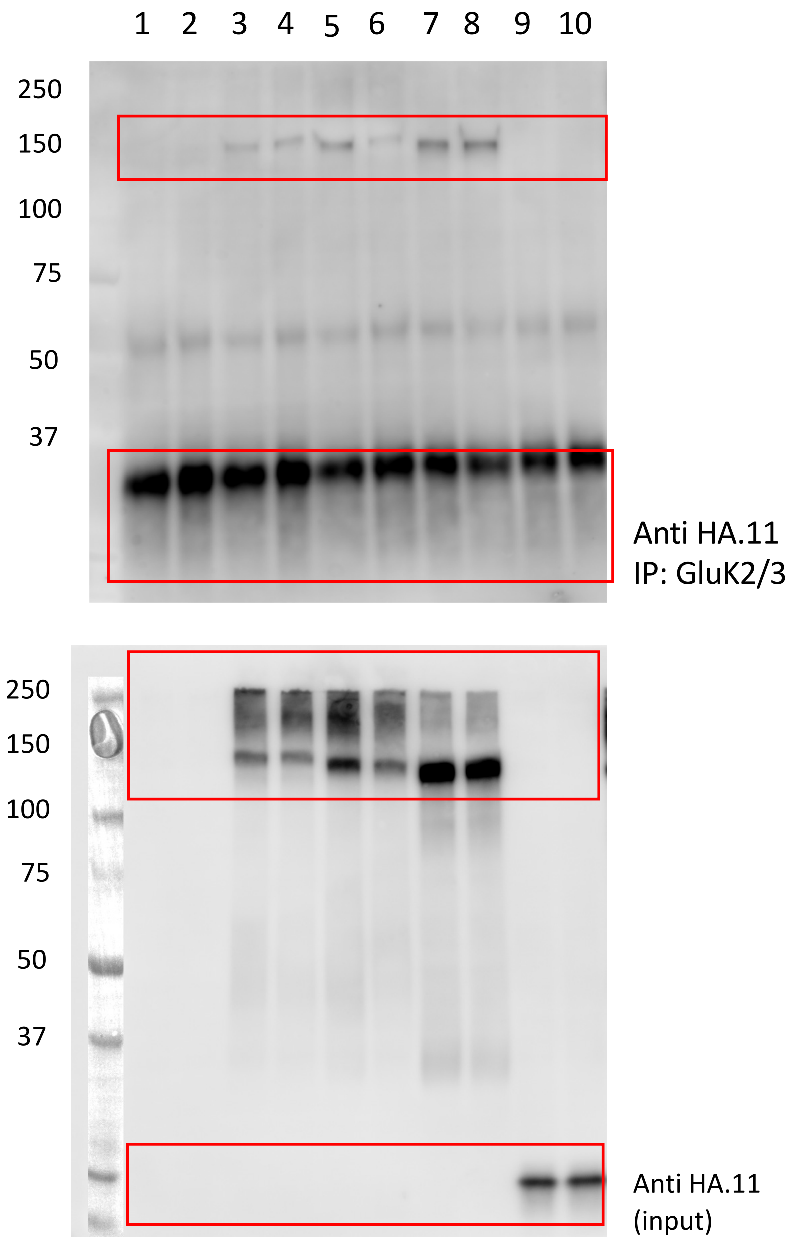

Supplement: Supplementary file 11 — Source Data for Figure 8 [file EMBJ-39-e103457-s009.pdf]
